# Insight into the evolution of *Vibrio vulnificus* biotype 3's genome

**DOI:** 10.3389/fmicb.2013.00393

**Published:** 2013-12-18

**Authors:** Vera Efimov, Yael Danin-Poleg, Nili Raz, Sharona Elgavish, Alex Linetsky, Yechezkel Kashi

**Affiliations:** ^1^Laboratory of Food Microbiology and Applied Genomics, Faculty of Biotechnology and Food Engineering, Technion – Israel Institute of TechnologyHaifa, Israel; ^2^Bioinformatics Knowledge Unit, Lorry I. Lokey Interdisciplinary Center for Life Sciences and Engineering, Technion – Israel Institute of TechnologyHaifa, Israel

**Keywords:** *Vibrio vulnificus*, evolution, biotype 3, genome, unique gene, gene transfer, environment

## Abstract

*Vibrio vulnificus* is an aquatic bacterium and an important human pathogen. Strains of *V. vulnificus* are biochemically classified into three biotypes. The newly emerged biotype 3 appears to be rather clonal and geographically restricted to Israel, where it caused an outbreak of wound infections and bacteremia. To understand the evolution of the bacterium's genome, we sequenced and analyzed the genome of biotype 3 strain VVyb1(BT3), and then conducted a microbial environmental survey of the hypothesized niche from which it probably evolved. The genome of this environmental isolate revealed higher similarity to the published biotype 1 genomes of clinical strains (90%) than to the environmental strains (87%), supporting the virulence of the biotype 3 group. Moreover, 214 of the total 5361 genes were found to be unique to strain VVyb1(BT3), having no sequence similarity to any of the known genomes of *V. vulnificus*; 35 of them function in DNA mobility and rearrangement, supporting the role of horizontal gene transfer in genome evolution. Interestingly, 29 of the “unique” genes had homologies among *Shewanella* species. In a survey conducted in aquaculture ponds in Israel, we successfully co-isolated *Shewanella* and *V. vulnificus* from the same niche, further supporting the probable contribution of *Shewanella* to the genome evolution of biotype 3. Indeed, one gene was found in a *S. algae* isolate. Surprisingly, molecular analysis revealed that some of the considered unique genes are harbored by non-sequenced biotype 1 strains isolated from the same environment. Finally, analyses of the biotype 3 genome together with the environmental survey suggested that its genome originated from a biotype 1 Israeli strain that acquired a rather small number of genes from other bacterial species in the niche, such as *Shewanella*. Therefore, aquaculture is likely to play a major role as a man-made ecological niche in bacterial evolution, leading the emergence of new pathogenic groups in *V. vulnificus*.

## Introduction

*Vibrio vulnificus* is a gram-negative halophilic bacterium which belongs to the family *Vibrionaceae*. It is a highly invasive human pathogen that is naturally found in marine and estuarine environments the world over (Strom and Paranjpye, 2000; Oliver, 2006; Jones and Oliver, 2009). Strains of *V. vulnificus* are classified into three biotypes based on biochemical and serological characteristics, and differences in host range. Biotype 1, isolated mostly from shellfish in coastal estuarine areas, is the most common group worldwide and is responsible for numerous clinical cases (Linkous and Oliver, 1999; Farmer, 2003). Biotype 2 was isolated from diseased eels and is rarely associated with human infections (Amaro and Biosca, 1996).

The newly emerged biotype 3 appears to be geographically restricted to Israel, where it caused an outbreak of wound infections and bacteremia among Israeli fish farmers and consumers of Tilapia fish (Bisharat et al., 1999). This novel group is responsible for nearly all clinical cases of *V. vulnificus* in Israel. Biotype 3 was found to be a clonal group that is clearly distinct from the other biotypes, possessing biochemical properties of both biotypes 1 and 2 (Bisharat et al., 2005; Broza et al., 2012). All biotype 3 isolates exhibited the same genotype in multilocus sequence typing (MLST) of 10 housekeeping and 5 conserved hypothetical genes (Broza et al., 2012). Analysis of 12 simple-sequence repeats (SSRs) loci, that are highly mutable regions, discriminated between its isolates (Broza et al., 2012), still demonstrating the low genetic diversity of this group in the tested loci. It has been suggested that the biotype 3 clonal hybrid evolved as a consequence of genome hybridization of two different and independent populations (Bisharat et al., 2005).

Even though *V. vulnificus* is a human pathogen, its evolution happens mostly in its natural environment—aquaculture fish ponds and Tilapia fish, which are the reservoir host of *V. vulnificus* biotype 3 in Israel (Bisharat et al., 1999). This emphasizes the importance of the local environment in shaping the genomic evolution of individual community members (Medini et al., 2008), including human pathogens. It is likely that the high organic biomass in aquaculture farms in Israel plays a major role as a niche for bacterial development, as well as providing selective pressure leading to new strains and groups, such as biotype 3 (Broza et al., 2012). Thus, we hypothesize that this aquaculture niche is the “melting pot” from which biotype 3 probably evolved. The main mechanism governing the development and emergence of new virulent strains in *V. vulnificus* is the high and frequent horizontal gene transfer in the *Vibrionaceae* (Quirke et al., 2006; Kim et al., 2011).

In the last decade, whole-genome sequencing has been used for the study of bacterial evolution (Jackson et al., 2011). This approach enables identifying large genomic rearrangements, including insertions, deletions, inversions, translocations and duplications (Bryant et al., 2012). Several comparative genomic studies have been conducted with *V. vulnificus* species to identify the specific genomic composition of different isolates (Quirke et al., 2006; Gulig et al., 2010; Morrison et al., 2012). The complete genome sequences of two *V. vulnificus* biotype 1 strains, YJ016 with CMCP6, were compared and showed large chromosomal regions that were unique to each. This suggested a role for DNA acquisition in increasing diversity and possible adaptability of the organisms to new and changing environments (Quirke et al., 2006). Sequencing of several biotype 1 and 2 genomes and subsequent comparative genomic analysis identified numerous genes that are common to the most virulent strains but are lacking from attenuated strains. These candidate virulence genes encode Flp pili, GGDEF proteins, and genomic island XII. Sialic acid catabolism was similarly identified as a potential contributory factor in molecular pathogenesis (Gulig et al., 2010). Recently, a pyrosequencing-based comparative study of six biotype 1 isolates identified 167 and 278 genes specifically associated with environmental and clinical genotypes, respectively (Morrison et al., 2012). The only available genome of biotype 3 was recently published by our laboratory, and provides a representation of this biotype due to the high clonality and very low diversity among strains revealed using molecular tools (Bisharat et al., 2005, 2007; Broza et al., 2007, 2012). This genome possesses a large number of genes that do not exist in the published biotype 1 genomes (Danin-Poleg et al., 2013). Analyzing this genome, and identifying the origin of new inserted elements and genes that are unique to biotype 3 in a bacterial population isolated from the aquaculture from which biotype 3 probably evolved, and tracing *V. vulnificus* in its natural ecological niche, should give an indication of horizontal gene transfer between the species. This, in turn, is expected to contribute to an understanding of the evolution of the human pathogen and provide a broad perspective of the emergence of new pathogenic strains.

## Materials and methods

### Bacterial strains and environmental sampling

Environmental samples were collected from Tilapia fish originating directly from artificial fish ponds, the fish store, and pond sediment in western Galilee, Israel, between 2009 and 2013 (during May–October). Fish samples included skin, gills and fins, and were isolated by a previously described procedure (Broza et al., 2009). Briefly, all samples were selectively enriched in alkaline peptone water with 4% NaCl, pH 6.9, overnight in duplicate, then plated on thiosulfate–citrate–bile salts–sucrose (TCBS) agar (HiMedia Laboratories, Mumbai, India) in 10-fold dilutions. Suspected *V. vulnificus* and *Shewanella* colonies (green and black, respectively) were further grown on chromogenic agar (CHROMagar Microbiology, Paris, France) for verification. *V. vulnificus* colonies were PCR-amplified for detection of the *vvh* gene (Broza et al., 2007). *V. vulnificus* isolates were identified by biochemical tests as previously described (Broza et al., 2012). Rapid crude DNA extraction from *V. vulnificus*- and *Shewanella*-suspected colonies was performed by ethanol-based technique as described previously (Buhnik-Rosenblau et al., 2013).

### Genome-sequence comparisons

The biotype 3 draft genome of VVyb1(BT3) (Danin-Poleg et al., 2013) was annotated using the RAST annotation server (Aziz et al., 2008). The complete genome was compared to the available *V. vulnificus* biotype 1 genomes of three clinical and three environmental strains, and similarity calculation was carried out by alignment of the whole genomes using MUMmer 3.0 software (Kurtz et al., 2004). The genome of VVyb1(BT3) was not compared to biotype 2 strains as there was no available genome sequence of biotype 2. In addition, genes “unique” to biotype 3 were identified by comparison of the annotated VVyb1(BT3) genes to the annotated genes of the published genomes of strains YJ016 (Chen et al., 2003) and CMCP6 (Kim et al., 2003) using stand-alone BLAST-2.2.23 (Altschul et al., 1990) and in-house scripts. In the second step, 435 “unique biotype 3 genes” were compared by BLASTn against the GenBank bacterial database.

### Detection of specific genes by PCR

Five genes with known function, “unique” to biotype 3 and present in *Shewanella* were selected for PCR amplification, and specific primers were designed based on available genomes of strains belonging to *V. vulnificus* biotypes 1, 3, and *Shewanella* targeting conserved regions (Table 1). The primers were used to generate ~200-bp fragments. The reactions were carried out in a Veriti 96-well thermal cycler (Applied Biosystems, Foster city, CA) as follows: 95°C for 3 min, 30 cycles of 30 s at 95°C, 30 s at the annealing temperature (52°C and 60°C), 90 s at 72°C, 10 min at 72°C, cooling to 12°C. PCR-amplification products were verified by 1.2% gel electrophoresis and observed by UV fluorescence. Both strands of the amplified products (Table 3) were also sequenced for verification, followed by multiple alignments (see further on).

**Table 1:** **Primers used for the amplification of “unique” biotype 3 genes**.

**Locus tag^a^**	**Forward primer (5′ → 3′)**	**Reverse primer (5′ → 3′)**
5128	AAACTTTCCAACCTCGTCGC	TACGAGGTTGTGGGCGATAA
5127	GTGTTACGATTGGGTCTCAGC	GCATTGACCACTCTGCTCTC
2770	ATCACGAGCGGTGAGTAAGG	AATGGCTGAACGAGTGGAAC
3563	TCGAAGTGATGAAGGGCAAC	AGCTCCTCCTCAATCCCATG
5118	AGGCGCCCGCGGCCGGGAAA	GCCAACTTCTTAGCAACCCG

a *Locus taq in VVyb1(BT3) genome*.

### Identification of *Shewanella*

Nucleotide sequence analyses of 16S rDNA and topoisomerase subunit B (*gyrB*) genes were performed to confirm *Shewanella* species identity (Yamamoto and Harayama, 1995; Nilsson et al., 2003). Fragments of the 16S rDNA gene and *gyrB* were PCR-amplified and the products were purified using a QIAquick PCR purification kit (Qiagen, Hilden, Germany). Purified DNA (20–50 ng) was sequenced on both strands using a BigDye terminator v1.1 cycle sequencing kit (Applied Biosystems) and loaded into an ABI 3130 genetic analyzer. Results were analyzed with SeqScape 2.5 software (Applied Biosystems) and DNA sequencing analysis 5.2 software (Applied Biosystems). Sequences of the isolates were compared with those of other *Shewanella* species in the GenBank database. Multiple sequence alignments were performed using CLUSTALW software (Thompson et al., 1994). The alignment files were used to evaluate genetic relationships among the strains by the unweighted pair group method with arithmetic mean (UPGMA) by MEGA 4.0 (Tamura et al., 2007). Bootstrap confidence values were based on 500 simulated dendrograms.

## Results and discussion

We recently published the first genome sequence of biotype 3 strain VVyb1(BT3), which afforded the opportunity to learn about the evolutionary process leading to the emergence of this new clonal pathogenic group of *V. vulnificus* (Danin-Poleg et al., 2013). The genome of strain VVyb1(BT3) exhibits features similar to those of published biotype 1 genomes and consists of two chromosomes and a plasmid (5.74 Mbp; 46.7% G + C content), including a total of 5361 protein-encoding genes. Whole-genome comparisons of strain VVyb1(BT3) to the available *V. vulnificus* biotype 1 genomes of three clinical and three environmental strains (Chen et al., 2003; Kim et al., 2003; Park et al., 2011; Morrison et al., 2012) showed that although VVyb1(BT3) is an environmental strain, it has higher similarity to the published genomes of clinical, rather than environmental, biotype 1 strains (~90% vs. ~87%, Table 2). This result, together with the high clonality of biotype 3 (Bisharat et al., 2005, 2007; Broza et al., 2009, 2012) and the fact that many of its isolates have a clinical origin, support the virulence of this group. However, more analysis is required to further support this assumption, as the compared environmental genomes are incomplete (presented as scaffolds and contigs).

**Table 2:** **Genome similarity as revealed by the whole-genome comparisons between biotype 3 strain VVyb1(BT3) and each of the six listed biotype 1 strains**.

**Genome**	**Similarity (%)**
CMCP6	90.4
YJO16	90.3
M06-24/O	89.2
E64MW	87.1
YJ1305	87.5
YJ7101	86.9

To better understand the special genomic features of biotype 3, a detailed analysis was performed. In the first stage, the genome of strain VVyb1(BT3) and the published *V. vulnificus* genomes of biotype 1 strains YJ016 (Chen et al., 2003) and CMCP6 (Kim et al., 2003) were compared. The analysis revealed a set of 435 genes that were absent in these biotype 1 genomes, suggesting that most of them are unique to biotype 3 (referring to *V. vulnificus* species) and may contribute to its virulence and environmental adaptation. Moreover, among the unique genes were those encoding proteins that might confer an advantage to biotype 3 against the microbial community in the environmental niche, such as the ParE-ParD toxin-antitoxin system, and against the host, such as hemoglobin-binding protein. In the second stage, the 435 genes were analyzed for sequence similarity to four more recently published *V. vulnificus* genomes (Morrison et al., 2012) using the BLASTn algorithm. Only half of the genes (214) were found to be unique to VVyb1(BT3) and had little or no similarity (filtering below 85% identity and 80% query coverage) to sequences in the known *V. vulnificus* genomes. This suggested that as more *V. vulnificus* genomes are compared, including those of biotype 2 strains, fewer genes will be recognized as “unique” to biotype 3.

**Figure 1:**
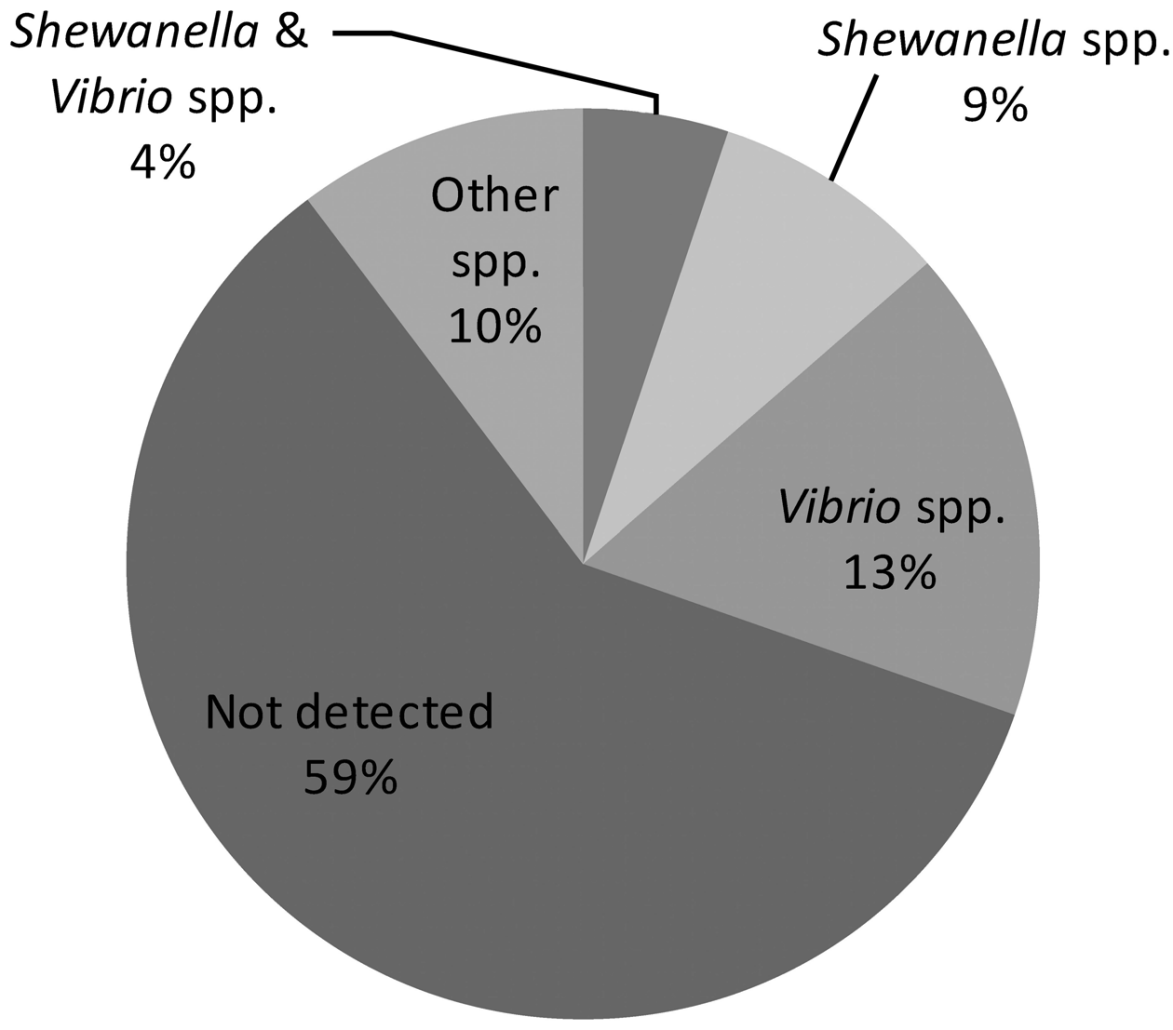
**Homology distribution of 214 “unique” genes of biotype 3 strain VVyb1(BT3) to other bacterial genes available in the GenBank database**. (Detailed information about the detected homologies is presented in supplementary Table S1).

The finding of “unique” biotype 3 genes led us to hypothesize that most of them are acquired horizontally from other bacterial species sharing the same ecological niche, such as other *Vibrio* and *Shewanella* species (see further on). Indeed, 35 of the annotated “unique” genes have functions in genome organization and DNA transfer, supporting a role for gene transfer in genome evolution. Therefore, in the last stage of the bioinformatics analysis, the BLASTn algorithm was used against the NCBI gene database to find homologs of the “unique” biotype 3 genes and to identify bacteria that might serve as donors for the gene transfer. The analysis revealed sequence similarity (>70% homology) of 87 genes to other bacteria (Figure 1; Table S1)**. As expected, due to the high horizontal gene transfer in the *Vibrionaceae* (Quirke et al., 2006; Kim et al., 2011), 37 genes showed homology to other *Vibrio* species: 10 genes were similar to *Vibrio parahaemolyticus*, 7 to *Vibrio harveyi*, 6 to *Vibrio fischeri*, and 14 to *Vibrio cholerae*. Interestingly, *Shewanella* shared a large number of genes (29) with the *V. vulnificus* biotype 3 genome. These two bacteria share similar environmental and clinical properties. The genus *Shewanella* also belongs to the family *Vibrionaceae* and is widely distributed in marine and freshwater environments (Hau and Gralnick, 2007). *Shewanella algae* and *Shewanella putrefaciens* are frequently found in non-human sources but are opportunistically pathogenic to humans (Tsai et al., 2008). Human infections include, among others, bacteremia, cellulitis (skin and soft tissue infection) and wound infection. The typical predisposing factor for infection with *S. algae* or *S. putrefaciens* is exposure to a marine environment with a skin lesion or skin trauma; other factors include the presence of a severe underlying debility, liver disease, or malignancy, and a compromised immune system (Oh et al., 2008). This all pointed to *Shewanella* as a possible gene donor that contributed to the formation of biotype 3, and called for an environmental survey.

Additional analysis revealed that 117 of the “unique” biotype 3 genes have no sequence similarity to any of the known sequences in the NCBI nr database (BLASTn: query coverage <30%, and *E* value >10^−10^), and most of them (86%) were annotated as hypothetical proteins. This suggested that these genes were acquired from new unknown or unsequenced species present in the marine niche, or underwent major changes during or after their integration into the genome. Results also emphasized that genome comparison in general is strongly dependent on the information available in the databases, and is limited and may not reflect the real picture, further calling for molecular analyses of the bacterial community in the habitat.

Environmental sampling was conducted to retrieve *V. vulnificus* isolates, as well as other bacteria that could serve as “candidate gene donors”—focusing on *Shewanella*—from the evolutionary niche from which biotype 3 probably emerged. Samples were taken from artificial fish ponds and fish stores in the western Galilee region of Israel, as most clinical cases in the last few years have been associated with fish aquaculture in this area (Broza et al., 2009). Hundreds of bacterial isolates were identified after selective enrichment from skin, gills and fins of Tilapia fish and sediment from a few different sample collections. Fish and sediment samples were found to be contaminated with *V. vulnificus*. Suspected *V. vulnificus* colonies were green on TCBS agar and showed the expected turquoise or white-colony phenotype on CHROMagar, indicating the presence of both biotypes 1 and 3 in the sample (Broza et al., 2009), with lower levels of the latter strains. Successful identification of *V. vulnificus* was confirmed by DNA amplification of the *V. vulnificus*-specific gene *vvh*. In addition, we found colonies that exhibit a typical *Shewanella* species phenotype, i.e., they were black on TCBS agar. Suspected isolates were found in sediment samples and were subjected to 16S rDNA and *gyrB* sequence analyses in comparison to *Shewanella* strains available from the GenBank database, confirming their identity as *Shewanella* species. Four isolates (Sh-4, Sh-6, Sh-9, and RD-4) presented different but rather similar sequence types in both genes. In a phylogenetic analysis, these isolates clustered together with *S. algae* strains and were separate from other *Shewanella* species, suggesting that the isolated colonies belong to *S. algae* (data not shown). Therefore, results showed successful co-isolation of *Shewanella* and *V. vulnificus* and further support the idea that *V. vulnificus* and *Shewanella* share the same environmental niche—aquaculture ponds in Israel—also supporting the probable contribution of *Shewanella* to the evolution of the biotype 3 genome.

Based on these findings, specific primers were designed to trace the presence of “unique” biotype 3 genes in *Shewanella* strains isolated from *V. vulnificus*'s natural environment by PCR amplification. Five different genes representing different cell pathways were checked: the ParE-ParD toxin-antitoxin system, two transcriptional regulators and a site-specific recombinase (Table 3). Six *V. vulnificus* strains, representing biotypes 1, 2, and 3, were used as controls. As expected, the tested genes were present in biotype 3 isolates. The five genes were not amplified in the two tested biotype 2 strains. Surprisingly, only one “unique” gene (2770) was present in one *Shewanella* strain (Sh-4), whereas the other four “unique” genes were not amplified in the four *S. algae* isolates, but were present in at least one biotype 1 isolate (Table 3). Analysis of the PCR-amplified products of the four tested loci showed sequence identity between biotype 1 and 3 strains. Moreover, 100% similarity was observed between *Shewanella* strain Sh-4 and *V. vulnificus* in the amplified fragment (243 bp) of the 2770 gene, supporting these genes' common origin. These results indicate that some of the genes considered to be unique to biotype 3 are present in biotype 1 strains isolated from the same environmental niche, suggesting that they are the possible origin for biotype 3 genes rather than *Shewanella* directly. Nevertheless, the isolated *Shewanella algae* and other yet unidentified *Shewanella* species in the environment might have served as gene donors for biotype 1 strains. Thus, we concluded that biotype 3 probably harbors unique genes acquired from other bacterial species, but the number of these is significantly less than the estimated 214.

**Table 3:** **PCR amplification results of five “unique” biotype 3 genes in *S. algae* and *V. vulnificus* isolates**.

**Locus tag ^a^**	**Gene function**	***V. vulnificus* isolates ^b^**						***S. algae isolates***			
		**yb82BT1**	**v252BT1**	**yb1 BT3**	**v247BT3**	**v209BT2**	**v212BT2**	**Sh-4**	**Sh-6**	**Sh-9**	**RD-4**
5128^c^	ParE toxin protein	+	+	+	+	−	−	−	−	−	−
5127^c^	ParD protein (antitoxin to ParE)	+	+	+	+	−	−	−	−	−	−
2770^c^	Transcriptional regulator	−	+	+	+	−	−	+	−	−	−
3563^c^	Transcriptional regulator	−	+	+	+	−	−	−	−	−	−
5118	Putative site−specific recombinase	−	+	+	+	−	−	−	−	−	−

a *Locus taq in VVyb1(BT3) genome*.

b *BT1, BT2, and BT3—biotypes 1, 2, and 3, respectively*.

c *Amplification products were checked for sequence similarity*.

This environmental survey, followed by the amplification of selected genes, indicated that the evolution of biotype 1 is a stepwise process that depends mainly on gene transfer within species as well as from other bacterial species sharing the same environment, thus leading to the constant creation of new strains. This is supported by a high diversity among biotype 1 isolates, as revealed by the SNP genotyping array of hundreds of *V. vulnificus* strains (Raz et al., unpublished), and by previous phylogenetic studies (Gulig et al., 2005; Broza et al., 2009, 2012; Sanjuan et al., 2011). According to this hypothesis, the continuous evolution and high horizontal gene transfer in *V. vulnificus* (Quirke et al., 2006; Kim et al., 2011) led to the creation of biotype 1 strains that are most similar to biotype 3, such as the clinical biotype 1 strain v252 that carried all five tested “unique” biotype 3 genes (Table 3) or other closely related strains isolated from the environment.

The genome analysis of biotype 3 showing its high similarity to other biotype 1 genomes, together with the presence of some considered biotype 3 “unique” genes in biotype 1 isolates and the SNP haplotype analysis (Raz et al., unpublished), leads to the conclusion that biotype 3's genome was created as yet-undiscovered event, based on the core genome of a biotype 1 strain that gained a rather small number of genes by horizontal gene transfer from its natural environment, leading to a change in biotype. Thus, we hypothesized that single episode of genome hybridization of two bacterial populations as suggested previously (Bisharat et al., 2005) may occur in *V. vulnificus*, however, it is less likely that this was the main event for biotype 3 creation. The new strain probably possessed better fitness under the selective pressure in the niche, as demonstrated by the clonal emergence of this biogroup. Similarly, serotype conversion was observed in *V. cholerae* when the specific DNA region responsible for this change was acquired from a non-*Vibrio* source (Stroeher et al., 1992). In addition, changes in gene expression might also cause the biochemical switch that led to the formation of new biotype. Data received from whole-transcriptome comparisons of the two biotypes by RNAseq performed by us (data not shown) and others (Bisharat et al., 2013) may provide an answer to this question.

To learn more about the creation of biotype 3, a full-genome comparison with a phylogenetically related biotype 1 strain should be performed, as this would enable focusing on a limited number of genes that separate the two biotypes. Furthermore, the high genetic diversity among *V. vulnificus* strains calls for an extensive multistrain full-genome comparison study together with high-throughput sequencing of whole bacterial communities in their natural habitat to fully understand the evolution of this human pathogen and the emergence of new virulent strains and biogroups.

### Conflict of interest statement

The authors declare that the research was conducted in the absence of any commercial or financial relationships that could be construed as a potential conflict of interest.
