# Supplementary material for: Insight into the evolution of Vibrio vulnificus biotype 3's genome
Source: Front Microbiol. 2013 Dec 18;4:393. doi: 10.3389/fmicb.2013.00393 (PMC3866513; doi:10.3389/fmicb.2013.00393)
Supplement: Supplementary file 1 [file DataSheet1.PDF]

Supplementary Material

Table S1: Homology of 87[1] 'unique' biotype 3 VVyb1(BT3) genes to other species available in GenBank database

Locus Taq[2]	Gene Function	Homology to Other Bacteria
1329	hypothetical protein	*Marinobacter hydrocarbonoclasticus*
1330	thioredoxin-dependent thiol peroxidase	*Psychromonas ingrahamii*
1331	thiol peroxidase, Bcp-type	*Vibrio* sp. EJY3
		Shewanella loihica
1332	hypothetical protein	*Vibrio* sp. EJY3
1333	hypothetical protein	*Vibrio* sp. EJY3
1334	hypothetical protein	*Vibrio* sp. EJY3
1335	MutL protein	*Vibrio* sp. EJY3
1469	mobile element protein	*Shewanella sediminis*
		Erwinia carotovora
		Legionella pneumophila
1511	MltA-interacting protein precursor	*Vibrio splendidus*
1598	hypothetical protein	*Coccidioides posadasii*
		Coccidioides immitis
1611	DNA sulfur modification protein DndB	*Pseudoalteromonas haloplanktis*
1612	cysteine desulfurase	*Enterobacter asburiae*
		Pseudoalteromonas haloplanktis
1613	Mrr restriction system protein	*Enterobacter asburiae*
21	membrane protein	*Vibrio fischeri*
		Vibrio furnissii
214	NTP pyrophosphohydrolase including oxidative damage repair enzymes	*Aliivibrio salmonicida*
		Vibrio fischeri
		Vibrio splendidus
216	DNA-cytosine methyltransferase	*Acinetobacter* sp.
		Cyanothece sp.
229	Adenine-specific methyltransferase	*Neisseria cinerea*
242	probable transposase	*Vibrio anguillarum*
		Vibrio cholerae
		Photobacterium damselae
2769	putative drug efflux protein	*Vibrio splendidus*
		Shewanella frigidimarina
		Shewanella amazonensis
2770	transcriptional regulator, LysR family	*Shewanella oneidensis*
		Photobacterium profundum

2771	2,4-dienoyl-CoA reductase	*Vibrio* sp. EJY3<br>*Shewanella oneidensis*<br>*Vibrio fischeri*
2772	N-ethylmaleimide reductase	*Vibrio* sp. EJY3<br>*Shewanella oneidensis*<br>*Photobacterium profundum*
2773	bifunctional protein: zinc-containing alcohol dehydrogenase; quinone oxidoreductase	*Vibrio* sp. EJY3<br>*Shewanella oneidensis*
3204	predicted lipase	*Vibrio* sp. Ex25
3563	transcriptional regulator, ArsR family	*Shewanella frigidimarina*
3589	beta-lactamase domain protein	*Vibrio parahaemolyticus*
365	hypothetical protein	*Vibrio parahaemolyticus*
366	protein ydhR precursor	*Vibrio parahaemolyticus*<br>*Shewanella sediminis*<br>*Photobacterium profundum*
367	transcriptional regulator, LysR family	*Vibrio parahaemolyticus*
379	RNA-directed DNA polymerase	*Pseudoalteromonas haloplanktis*
386	hypothetical protein	*Shewanella putrefaciens*
3937	transcriptional regulator, HxlR family	*Vibrio parahaemolyticus*<br>*Shewanella loihica*
3938	hypothetical protein	*Shewanella sediminis*
393	hypothetical protein	*Vibrio harveyi*
394	repressor-like protein	*Vibrio cholerae*
395	hypothetical protein	*Vibrio cholerae*<br>*Vibrio tapetis*
396	hypothetical protein	*Vibrio anguillarum*<br>*Vibrio furnissii*
401	mobile element protein	*Vibrio anguillarum*<br>*Listonella anguillarum*<br>*Vibrio harveyi*
4275	mobile element protein	*Vibrio harveyi*<br>*Listonella anguillarum*<br>*Photobacterium profundum*
4325	hypothetical protein	*Vibrio harveyi*<br>*Vibrio tapetis*
5031	pseudaminic acid biosynthesis protein PseA, possible Pse5Ac7Ac acetamidino synthase	*Roseburia hominis*
5033	Imidazole glycerol phosphate synthase cyclase subunit	*Roseburia hominis*
5034	N-Acetylneuraminate cytidylyltransferase	*Vibrio fischeri*

5036	N-acetylneuraminate synthase	*Vibrio fischeri*<br>*Aeromonas punctata*<br>*Photobacterium profundum*
5102	hypothetical protein	*Photobacterium damselae*
5104	IncF plasmid conjugative transfer pilus assembly protein TraH	*Photobacterium damselae*
5116	IncF plasmid conjugative transfer protein TraN	*Shewanella* sp. W3-18-1<br>*Photobacterium damselae*<br>*Proteus mirabilis*
5118	putative site-specific recombinase	*Shewanella oneidensis*<br>*Shewanella putrefaciens*<br>*Vibrio cholerae*
5120	hypothetical protein	*Yersinia pestis*
5121	mobile element protein	*Vibrio anguillarum*<br>*Listonella anguillarum*<br>*Xenorhabdus nematophila*
5122	hypothetical protein	*Vibrio* sp. 23023
5123	mobile element protein	*Vibrio harveyi*<br>*Marinomonas* sp.
5124	mobile element protein	*Vibrio harveyi*<br>*Marinomonas* sp.
5125	hypothetical protein	*Vibrio harveyi*<br>*Vibrio harveyi*
5126	putative site-specific recombinase	*Vibrio cholerae*<br>*Shewanella oneidensis*<br>*Photobacterium damselae*
5127	ParD protein (antitoxin to ParE)	*Vibrio cholerae*<br>*Shewanella putrefaciens*<br>*Shewanella oneidensis*
5128	ParE toxin protein	*Vibrio cholerae*<br>*Shewanella oneidensis*<br>*Shewanella putrefaciens*
5131	hypothetical protein	*Shewanella* sp. MR-7
5132	TrwC protein	*Shewanella* sp. MR-7
5133	IncF plasmid conjugative transfer protein TraD	*Shewanella* sp. MR-7
5134	hypothetical protein	*Shewanella* sp. MR-7
5135	hypothetical protein	*Pectobacterium wasabiae*<br>*Halothiobacillus neapolitanus*<br>*Advenella kashmirensis*
5136	replication P family protein	*Shewanella* sp. MR-7
5139	hypothetical protein	*Shewanella* sp. MR-7
5140	hypothetical protein	*Shewanella* sp. MR-7

5142	hypothetical protein	*Vibrio parahaemolyticus*<br>*Vibrio* sp. EJY3
5143	hypothetical protein	*Vibrio parahaemolyticus*<br>*Vibrio anguillarum*
5144	hypothetical protein	*Vibrio parahaemolyticus*
5263	hypothetical protein	*Shewanella* sp. MR-7
5264	hypothetical protein	*Shewanella* sp. MR-7
5267	hypothetical protein	*Shewanella sediminis*
5268	hypothetical protein	*Shewanella sediminis*
5269	hypothetical protein	*Shewanella* sp. MR-7
619	hypothetical protein	*Photobacterium damselae*
656	hypothetical protein	*Vibrio cholerae*<br>*Vibrio anguillarum*<br>*Photobacterium damselae*
657	hypothetical protein	*Vibrio anguillarum*<br>*Vibrio cholerae*
658	hypothetical protein	*Vibrio anguillarum*<br>*Vibrio cholerae*<br>*Photobacterium damselae*
785	Retron-type RNA-directed DNA polymerase	*Citrobacter rodentium*<br>*Dickeya zeae*<br>*Dickeya dadantii*
843	hypothetical protein	*Vibrio cholerae*<br>*Vibrio splendidus*<br>*Vibrio metschnikovii*
846	hypothetical protein	*Vibrio cholerae*
855	HipA protein	*Vibrio anguillarum*<br>*Vibrio cholerae*

[1] Addition six ORFs from phage origin, having a role in DNA transfer, were included ‘Other species’ section in Figure 1.
[2] Locus taq in VVyb1(BT3) genome.
